# Time poverty of families of children with 22q11DS: a healthcare professional perspective

**DOI:** 10.1186/s12887-026-06836-0

**Published:** 2026-05-06

**Authors:** Sophie Ayoub, Eva De Clercq, Sandra Meier, Ann Swillen, Cheryl Cytrynbaum, Luzius A. Steiner, Holly Carbyn, Bernice S. Elger

**Affiliations:** 1 Institute for Biomedical Ethics, University of Basel, Basel, Switzerland; 2 Department of Psychiatry, Dalhousie University, Halifax, NS Canada; 3 IWK Health Centre, Halifax, NS Canada; 4 Center for Human Genetics, UZ Leuven, Leuven, Belgium; 5 Department of Human Genetics, KU Leuven, Leuven, Belgium; 6 Division of Clinical & Metabolic Genetics, Department of Genetic Counselling, the Hospital for Sick Children, Toronto, Canada; 7 Department of Molecular Genetics, University of Toronto, Toronto, Canada; 8 Department of Anesthesiology, University Hospital Basel, Basel, Switzerland; 9 Department of Clinical Research, University of Basel, Basel, Switzerland; 10 Center for legal medicine, Faculty of Medicine, University of Geneva, Geneva, Switzerland

**Keywords:** Rare disease, Equity, Time management, Coordination of care

## Abstract

**Introduction:**

22q11 deletion syndrome (22q11DS) is the most common microdeletion in humans, leading to a wide range of variable clinical manifestations that can affect any part of the body. Most individuals with 22q11DS are diagnosed during childhood and the responsibility of care falls on their primary caregivers, usually their parents, who need to balance the needs of all family members including themselves. This affects parental wellbeing. This study investigates the perspective of healthcare professionals (HCPs), in Europe and Canada, on the parents’ struggle with time invested in caring for their children, factors contributing, the impact of this responsibility and some strategies on how to support the families.

**Methods:**

This interview study was part of a larger research initiative aimed at improving the psychosocial well-being of individuals with 22q11DS and their families. The qualitative component concentrated on gathering insights from HCPs involved in patient care. We conducted qualitative content analysis after transcribing the semi-structured interviews. The research question focused on time poverty within families caring for a child with 22q11DS from the perspective of HCPs.

**Results:**

The 20 HCPs interviewed came from diverse professional backgrounds, but all had clinical experience with children with 22q11DS. Our analysis of the data identified three primary themes, centered on time poverty of families caring for a child with 22q11DS. Centralization of care and insufficient coordination were the main modifiable reasons for the time struggle, affecting the families, especially mothers and families living in rural areas. Strategies proposed to mitigate this issue included improved coordination and decentralization of care, and digitalization.

**Conclusions:**

Time poverty for families with a child with 22q11DS may result from fragmented and uncoordinated medical care, and centralized care disconnected from local care. Better communication between different HCPs, local and central, and a robust support system could enhance these families’ well-being and assure an equal distribution of care services without gender bias or exclusion of rural underserved populations.

**Supplementary Information:**

The online version contains supplementary material available at 10.1186/s12887-026-06836-0.

## Introduction

22q11 deletion syndrome (22q11DS) occurs when a small piece of the long arm of chromosome 22 is missing. Although a rare disease (RD) (affecting less or equal to 1/2000) [1], It is the most frequent microdeletion in humans [2], with an incidence of 1/2148 [3]. The clinical manifestations are broad and can affect any bodily system. Common features include congenital heart defects, palatal anomalies and immune deficiencies. Additional clinical findings include a broad range of neurodevelopmental and psychiatric disorders [4–6]. There is an extensive clinical variability with symptoms varying from mild to severe but most individuals with 22q11DS require a multidisciplinary approach and coordinated professional care throughout their lives [4–6].

Like most patients with rare disease (RD), the majority of individuals with 22q11DS are often diagnosed in childhood [7]. Thus the responsibility of care predominantly falls on their primary caregivers, most often their parents, who must meet their specific needs [8]. Caring for a child with 22q11DS can be overwhelming and demands significant attention [8, 9], as parents must juggle their roles as both caregivers and parents [10]. In addition, a range of unique challenges complicate the situation, particularly the uncertainty [11], psychological, medical, and social consequences that arise from inadequate knowledge about the condition in both healthcare and social settings [12]. The challenges are compounded by the lifelong changing nature and rarity of the condition [13, 14], as well as the limited number of specialized clinics, professionals and access to services [15].

Parents of children with RDs, particularly those with complex medical needs like 22q11DS [16], face great difficulty in managing their duties due to time constraints [10, 17]. Balancing their personal needs, their marital needs and the care of their healthy children with those of their child affected by an RD is a challenging task and a big concern [15, 18]. Along these lines, in their study exploring the experiences of caregivers of children with RDs, Witt et al. found that children and adolescents with RDs, along with their parents, view time as a significant barrier that burdens their daily lives due “time-intensive requirements” including medical appointments, housework, family obligations, and child care [19]. An exploration of the needs of caregivers of family members with 22q11DS by Cosman et al. also identified lack of time as a barrier to getting psychological help for themselves when needed and a limiting factor to the support of caregivers’ needs and concerns [20].

Since 22q11DS is one of the more common rare diseases, numerous syndrome-specific family support networks exist, and considerable information is available regarding the medical and educational needs of affected individuals. However, how families manage their time remains largely underexplored, particularly from the perspective of healthcare professionals (HCPs) working in the fields of 22q11DS and RDs. In fact, most papers on 22q11DS with professionals discuss the medical management of the symptoms [21, 22], focusing on the diagnosis and the clinical perspective, even though the impact of 22q11DS goes far beyond clinical facets and touches on every aspect of the caregivers’ lives [16]. Studies have also shown that families living with an RD appreciate the experts’ knowledge and seem more satisfied with the healthcare system when HCPs are more knowledgeable [23–25].

For these reasons, we decided to investigate how HCPs perceive parents’ struggles with managing time while caring for their children. The goal of the study is to explore the reasons behind families’ time poverty, their reactions to it, and potential strategies to alleviate this burden from the perspective of HCPs.

## Methods

This interview study is part of a larger research project on the psychosocial well-being of children with 22q11DS and their families.

The qualitative component of the study focuses on the caregiving experiences of parents and HCPs. This paper presents findings from interviews with HCPs. A second part of the study involved interviews with families, focusing on different research questions; those results will be reported separately. While the current paper presents qualitative findings, a separate quantitative study addressed related questions within the same population and will not be referred to in this paper anymore.

*Note from the authors: Another manuscript on the same sample of HCPs can be found in the literature. However*,* each paper was developed to provide an in-depth analysis of a different aspect of the data. Together*,* they offer complementary insights into the broader project* [23].

### Data collection

#### Ethics approval

The project was approved by the Ethics Committee of the University of Basel (reference number 119). The study was conducted in compliance with the protocol, the current version of the Declaration of Helsinki, the ICH-GCP, or ISO EN 14,155 (as far as applicable) as well as all national legal and regulatory requirements. The data were stored in accordance with the General Data Protection Regulation (GDPR) on a secure university server and were only accessible to the research team.

#### Sample

HCPs were eligible if they were working in Europe or Canada and had at least 1 year of experience caring for children aged 3-15years with 22q11DS and their families. The recruitment consisted of purposeful sampling (*N* = 2) through clinicians who collaborated on the project and snowballing (*N* = 10). Another round of recruitment consisted of contacting 21 board members from leading 22q11DS organizations (22q11europe, 22q, and 22q Society) via email. The email sent provided an overview of the research project and invited them to participate in interviews. Of the 11 professionals who replied, some met the eligibility criteria and were interviewed (*N* = 6). These interviewees also provided contact information for two additional potential participants who met the inclusion criteria and were interviewed.

#### Interviews

The interview process started with a pilot interview in October 2022, and continued through April 2023. All interviews were conducted by SA, a female medical doctor doing a PhD in Bioethics and qualified in qualitative research techniques. HCPs were sent an information sheet and a written informed consent that they returned via email after signing. All interviews were semi-structured and done in English via the digital platform Zoom. Before the start of the interview, participants were re-informed about the purpose of the study, the risks and benefits and consented again orally to participate. The respect of privacy and confidentiality were also discussed. The development of the interview guide was guided by existing literature, shaped through discussions within the research team and 2 pilot-interviews and covered topics focusing on HCPs’ caregiving experience, the perceived needs of families, available support services, the perceived impact of the condition on families, and the coping experience of HCPs. HCPs were additionally asked about their perception of the future and the role of digitalization (i.e. video consultations). In this paper, we will focus on the perceived needs and available support services including digitalization. The rest of the topics will be discussed in other papers.

### Data analysis

**Fig. 1:**
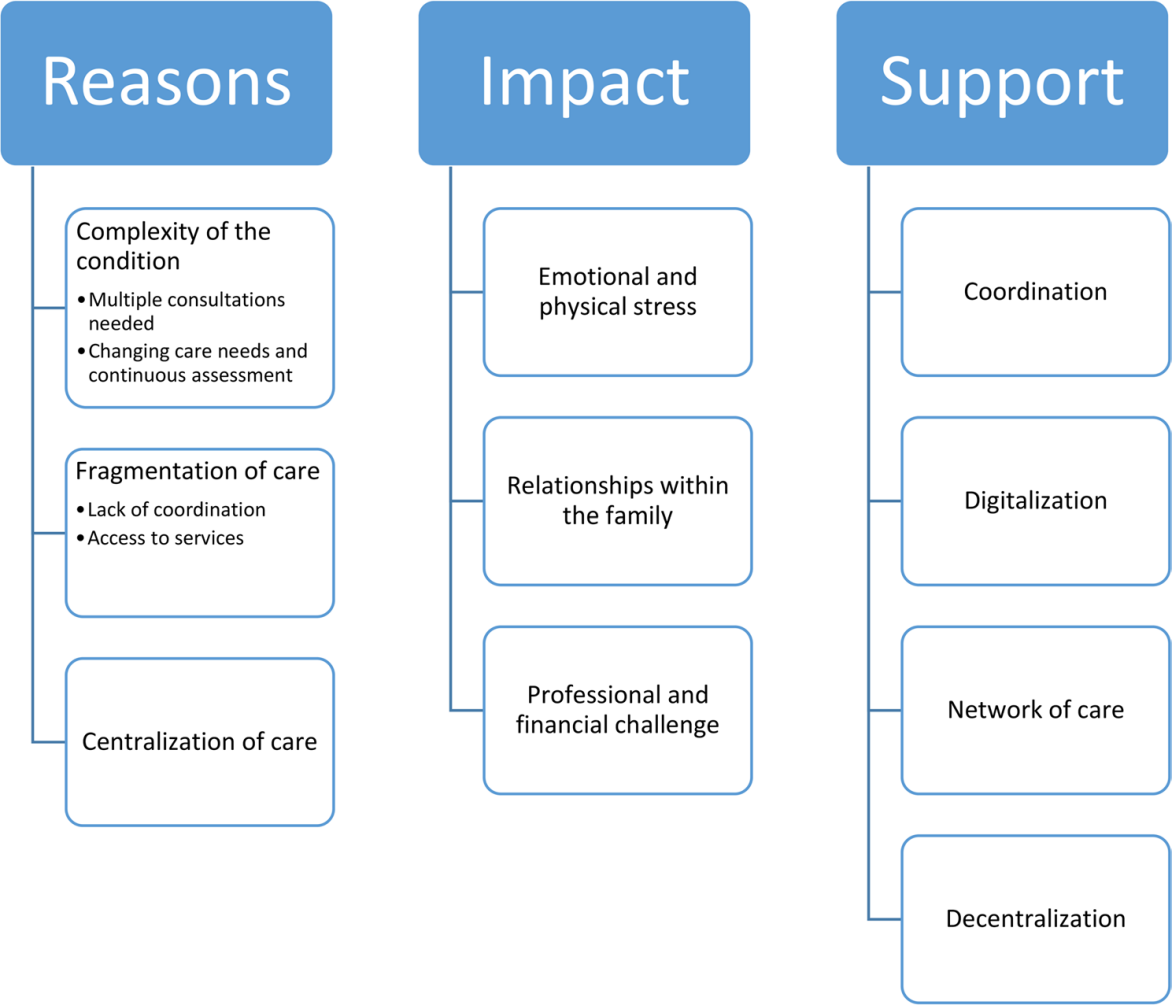
Themes and subthemes

All interviews were recorded upon consent, and transcribed using the qualitative analysis software MAXQDA 24 to support structured data analysis. All personal information was de-identified following verbatim transcription to maintain anonymity and respect for privacy and confidentiality. Data were analyzed using Graneheim and Lundman (2004) [26] qualitative content analysis with an inductive category approach, therefore, the concepts were derived from the data [27]. The research question focused on time poverty within families caring for a child with 22q11DS from the perspective of HCPs. This study was guided by a constructivist paradigm, acknowledging that reality is socially constructed and multiple interpretations can coexist. The analysis focused on manifest content [26]. Initial coding was conducted by two researchers SA and EDC, who engaged in reflexive discussions to explore interpretations and ensure analytical depth, organizing the data into main categories and sub-categories (see Fig. 1). In line with a constructivist approach, our focus was on shared meaning-making through dialogue rather than intercoder reliability. Furthermore, consistent with a constructivist paradigm, we drew on Lincoln and Guba’s trustworthiness criteria—credibility, transferability, dependability, and confirmability—to ensure rigor in our qualitative analysis [28]. We attained credibility, transferability and dependability through researcher triangulation and thick description. Reflective debriefing by the researchers during the collection and analysis of data allowed us to achieve confirmability.

## Results

In this research, we conducted interviews with 20 HCPs, consisting of 16 females and 4 males. The interviews lasted between 40 and 77 min in length. Participating HCPs came from diverse disciplines: 2 genetic counselors, 4 psychologists, 2 psychiatrists, 2 ENT (ear, nose, and throat) surgeons, 3 pediatricians, 4 speech-language pathologists, 1 clinical immunologist, 1 nurse-coordinator, and 1 occupational therapist. Most of these professionals had over a decade of experience and were affiliated with a 22q11DS clinic, located either in Canada or Europe (see Table 1).

**Table 1:** Demographics of interviewed healthcare professionals

HCP	Sex	Specialty	Years of experience with 22q11	Number of children with 22q11DS in care per year
HCP1	F^(1)^	Genetic counselor	21–25 years	100–120
HCP2	F	SLP^(3)^	35–40 years	70
HCP3	F	Psychologist	25–30 years	450
HCP4	F	Psychologist	1–5 years	20
HCP5	M^(2)^	ENT surgeon	21–25 years	24–30
HCP6	F	SLP	21–25 years	40–45
HCP7	F	Nurse coordinator	21–25 years	NA
HCP8	F	Genetic counselor	15–20 years	60–120
HCP9	F	Child psychiatrist	1–5 years	24
HCP10	F	ENT surgeon	10–15 years	60–120
HCP11	M	Pediatrician	35–40 years	450 in total
HCP12	F	Pediatrician	25–30 years	60–120
HCP13	M	Clinical immunologist	20–25 years	2–3
HCP14	F	Psychologist	10–15 years	60
HCP15	M	Psychiatrist	20–25 years	20 in total
HCP16	F	SLP	10–15 years	100
HCP17	F	Occupational therapist	5–10 years	2–3
HCP18	F	Psychologist	15–20 years	70–80
HCP19	F	Pediatrician	5–10 years	100
HCP20	F	SLP	20–25 years	Variable

(1) Female; (2) Male; (3) Speech-language pathologist

Our analysis of the data identified three primary themes, all centered around time poverty of families caring for a child with 22q11DS from the perspective of HCPs, but addressed from different aspects: (1) Why are families caring for a child with 22q11DS time-poor? (2) What is the impact of time poverty on families? (3) How to support families struggling with time poverty?

### Reasons

The majority of HCPs identified challenges encountered by families in terms of time while caring for a child with 22q11DS. They identified different reasons for this time poverty: (1) the nature of this condition, (2) the fragmentation of care and (3) the centralization of the healthcare system (see Table 2).

#### Complexity of the condition: 22q11DS, a long and changing journey

All the experts described the condition as complex and broad as it can affect almost any bodily system and can vary from life-threatening to very mild. In addition to physical health issues, many patients also experience intellectual disabilities. The combination of both these physical and cognitive impairments had a significant impact on their daily activities and required a lifelong follow-up. Therefore, individuals with 22q11DS often need appointments with different specialists for long periods, which is very time-consuming for families. Moreover, due to the possible onset of new health and neurodevelopmental problems over time, many HCPs emphasized the need for ongoing assessments and regular check-ups, further adding to the time burden for parents.

#### Fragmentation of care

Our participants emphasized the importance of effective coordination for the numerous medical appointments patients need to attend. This coordination was not only related to regular consultations but also to the chronological order of the needed procedures by health priority. They also endorsed that outside of a specialized 22q11DS clinic, there is no dedicated individual to facilitate coordinated care, and HCPs, genetic counselors for example, try to take on this role to synchronize appointments for their patients.

Many HCPs perceived how the fragmentation of care can also be related to the challenge of knowing what services exist and how to find them. They recognized that the system often complicates access to services, making it challenging for parents to identify and secure the support they need, even when excellent resources are available. The assistance from professionals can help but is not always sufficient. One participant mentioned that identifying some services might be difficult for parents and for healthcare providers as well, even geneticists.

#### Centralization of care

Almost all HCPs mentioned that multidisciplinary teams and experts in the field are usually located in specialized centers in big cities and centers. It can be thus challenging for some families, particularly those living in rural areas, to travel for their medical appointments. The journey was seen as challenging for working parents, both in terms of time and financial strain, which could hinder their ability to access necessary services. The professionals emphasized the complexity of commuting long distances for regular appointments especially when follow-ups with local healthcare providers are not an option. However, HCPs noted that when families live close to the center, centralization causes no problem.

**Table 2:** Reasons for time poverty

1.1.Complexity of the condition
HCP2 (speech-language therapist): It’s such a multisystem disorder that many families are dealing with. They’re dealing often with cardiac issues. These children tend to be a little sicker than other children, so speech is just one added layer (…) I think for some families it is a little bit overwhelming dealing with so many different professionals and so many different layers of dysfunctions (…)
HCP9 (psychiatrist): Many parents struggle with mental health and anxiety and burn out and administratively, it’s unbelievable how many appointments and meetings are needed to coordinate kids with medical and developmental complexities (…) I have families where one parent had to stop work because they have a medical appointment twice a week so what can they do?
HCP6 (speech-language therapist): One of the things with the 22q population from a speech perspective is the motor planning aspect that goes along with their speech, that does not get remediated quickly, for many of these kids it’s a long road for speech therapy, (…) 10 sessions of speech therapy over the course of the year is not going to do it, this particular child there needs to be much more (…)
HCP7 (nurse coordinator): It’s so much organizing what has to be done and worrying and it’s definitely a burden of care because it’s a more complex medical diagnosis requiring ongoing assessments and changing of care.
1.2.Fragmentation of care
HCP3 (psychologist): I think what parents need and that is what I hear all over the world, that the care is still not coordinated, integrated and it’s quite separated or fragmented.
HCP2 (speech-language therapist): (…) They (geneticists) ‘re constantly calling to say hey (name of child) is coming next Tuesday and we noticed that he is supposed to see speech and no one’s followed up with him- I’m just giving an example- no one’s followed up with him in 3 years, can we get him in because he’s coming from far away. They’re the gate keepers in a way and then we also follow some kids within our own system and again we do our best to try and coordinate appointments (…)
HCP9 (psychiatrist): (…) I do think they are expected to navigate the care much more than the average parent, and that itself is a job. Like often, it becomes a full time job to navigate care for kids if they have multisystem complexities and yeah there are programs in (city) for complex care kids that come with care navigators, but only a few of the 22q11DS have met the threshold for that level of complexity for that program. So really parents end up doing all of that case navigation, appointment booking, and it’s a lot.
HCP13 (clinical immunologist): Of course, if the child is identified as disabled, there would be some sort of financial and social support that is provided by the state but you know they will have to navigate the system and that can be quite complex. But can be guided by both the community pediatrician and also by the organization.
HCP8 (genetic counselor): Part of what we do is we help them navigate all the community services that are around, some of them are there, it’s just there’s no centralized way of accessing them, so you have to know about them. If the child is struggling with mental health, I am aware of some of great resources that I can connect parents to, but I have no way of identifying every single potential resource and neither do they.
1.3.Centralization of care
HCP5 (ENT surgeon): I think it depends on the family and also geography. So the problem is that (province) is probably about 10 times bigger than Switzerland, it’s quite large and people will have to travel long distances, and the community supports in terms of speech therapy can be a challenge and so to me that’s the biggest problem the families will have if they’re coming from a long distance. If they’re close by then that’s not really a major issue (…)
HCP7 (nurse coordinator): I think the challenging aspect is for families who don’t live near a big center like our center and they don’t have access to the coordinated care by HCPs that really understand what these issues are and how they interrelate and that goes for different health issues. And the further you live away the harder it is for families to access care and it is hard for families to travel to big centers, you know they’re working, there’s the cost (…)
HCP14 (psychologist): yeah I guess one of the difficulties like I said earlier is the distance, that some of the parents have to travel to see you. So in terms of psychological, many of the district hospitals also have psychologists but because some people would come to the multidisciplinary clinic, they would then want to come to see the psychologist that had been in that clinic and of course logistically it can be difficult for families.
HCP19 (pediatrician): I think something that is special to our center that it’s a cardiology center sometimes they see us when they are admitted to the cardiology department and then we have to tell them that unfortunately there is no 22q team in your area.
HCP6 (speech-language therapist): Then also accessing services for patients especially those who live in sort of remote areas where they may not have access to speech therapy or the frequency of therapy that would really benefit patients with 22q, that can be frustrating sometimes (…) so them being able to make the trip and come to the hospital, those are the main challenges.
HCP11 (pediatrician): Well again, that would be if they are referred for something like speech therapy and they live in (city), then they would get specialists plugged in in a very early stage and that’s because they would come to the center. But there is limited number of centers, and the (clinic) that runs 22q services, there they would be seen by speech therapy very rapidly because they have the resources and they know what they are dealing with.

### Impact

All our participants acknowledged that caring for a child with 22q11DS impacts families in multiple ways. They observed that spending a lot of time attending to the medical and developmental needs of their children can reduce their opportunity to follow a study or a career path and can cause higher emotional and physical stress that can lead to challenging family dynamics (see Table 3).

**Table 3:** Impact of time poverty

2.1.Emotional and physical stress for the parents
HCP15 (psychiatrist): Like 22q11DS, we know that parents who have a child who has a neurodevelopmental disorder, mothers have less income, they get less chances, less opportunity to study, less opportunity to work, they have a higher rate of dementia when they are old, the mothers of developmentally disabled children they are at a higher risk of developing dementia when they are old because of chronic stress of their entire life, there are more divorces, much more divorce in families who have a developmental disorder.
HCP3 (psychologist): (…) The parents you know they’re resilient but what is the level of their resilience? is it still doable for them? because they get a lot of advice, you should go to speech language therapy, you should do physiotherapy, he has to have 10 appointments. And then of course parents say I do not know where to start, I do not even know to come through the day. I have another child or I have to work, I have bills to pay. So it’s a lot for them (…)
HCP2 (speech-language therapist): Listen, I think it’s challenging with any child who has issues whatever those issues are, whether medical issues or psychosocial issues or behavioral issues. It’s challenging to navigate that (…)
HCP4 (psychologist): Yeah, I mean some parents say look I’m worn out physically, some are struggling just to physically care for themselves, some have chronic health problems and they talk about the fact that they have to go to therapy themselves, they have their own health problems and sometimes just the physical burden of caring for their child can make their health problems worse. So some kids need a lot of physical care if they’re more disabled, some are just you know you’re busy all the time, and it just wears you down physically (…) For them it’s also economic, it’s social, it’s language barriers and they feel isolated.
HCP1 (genetic counselor): When you get to the early grades, a neurotypical child you might leave them on their own to do their homework, but a child with 22 and learning disability needs ongoing support. So it can be very exhausting. I know a lot of parents who have been just completely burnt out from having to support their children in school because they’re just not getting enough in school and it’s exhausting (…) so there’s just such a storm of so many different issues and dealing with the emotional and the physical it is absolutely exhausting. And they put themselves last a lot of them right? and so you take care of your children and then you forget that you need to replenish your own tank in order to be able to care for your children.
HCP3 (psychologist): There is also the parents, this is the part about balance in your life how much burden can they take? and what is their resilience? so it’s too much stress for everyone in the family but the parents particularly, a high level of stress for mothers, fathers also, but I think still for a lot mothers (…) the child has to be so much in the clinic so they are often in a lot of stress, physical stress and emotional stress.
HCP14 (psychologist): yeah so this is only my experience but it is the moms struggle more than the dads definitely, and saying that, maybe that is not true actually, I was going to say maybe I’ve seen more of the moms than the dads but I’ve seen a lot of dads as well of children with 22q. I would say moms seem to, I don’t know what’s more difficult, I don’t know if it’s because they take upon themselves to try and support with all the things that are difficult, that are tricky, so they intervene with school things (…) and what I see with most dads, again it’s not a generalization, but all the dads I have in mind are much more practical in what they do: so let’s take one day at a time, this might be fine, let’s not worry about this, he’ll find friends, he’ll find his own pace in the world. I would say few moms I’ve seen have been quite depressed because they struggle to manage and then at the expense of other family members, the siblings they get probably left to manage a bit on their own.
2.2.Unsettled family environment
HCP1 (genetic counselor): There is also of course family dynamics when sometimes parents have different perspectives and different approaches and different degrees of acceptance of the child’s special needs, navigating those marital relationships. And if there are other siblings in the family sometimes for parents, balancing the needs of -let’s call him the special needs child vs. neurotypical child- that’s also difficult to juggle. And sometimes the neurotypical children do not understand why the other child is getting so much attention and it creates other familial stressors. I have met with a number of siblings to talk to them about the diagnosis to try and help them understand because you know if you’ve got a 12-year-old, they don’t understand necessarily why their 15-year-old sister who is older needs so much support (…)
HCP4 (psychologist): So for the families I think we talk to them about the importance of respite care, of being able to spend time with them as a couple, or spend time with their other kids. Quite often, they say look at all my attention goes to my child with 22q and I have other kids and I feel really bad about the fact that they don’t get a lot of my time and the other kids are complaining, saying you don’t spend time with me, you don’t care about me.
HCP12 (pediatrician): Maybe not very easy to say but it has impact on the family life, it has because it is a lot of work, it is more than a job when you have a child like this, and you need to help them and they need to go to specialists often, and it affects the siblings because they need a lot of time. And I think how much it affects the relation I cannot say, but I really think it does because we know there are often more divorces and so when parents have a child with a difficult condition.
HCP18 (psychologist): (…) The fact that the parents are caring for the child who has some difficulties and how to balance that with the other brothers and sisters. They put a lot of attention and effort on accompanying this child to the doctor, to the speech therapist and so on, spend a lot of time doing the homework and they do that less for the other kids (…) there can be a lot of frustration also from the siblings. For the parents, one of the challenges is also the balance between the two parents, so who is taking the burden in a way of also yeah being there for the child, (…) and often what we see is it is a bit imbalanced between the two parents and one is taking the burden a bit more than the other one (…)
HCP17 (occupational therapist): (…) I work with some siblings who are caregivers too, it’s like I think it depends, I see two sorts of directions that could go sometimes in terms of the extremes of it, I have a lot of siblings who are nurses themselves, like they go into some sort of helping profession and it’s been like something that really shaped their life and it’s like a particular direction around helping, I’ve seen some siblings feel a lot of resentment or exhaustion or caregiver fatigue almost or burn out themselves, from the level of stress that’s going on around the house, particularly if it’s like a psychiatric or behavioral issue.
HCP19 (pediatrician): I think that the families with children who have intellectual disabilities and neuropsychiatric disorders have of course a difficult situation. I would say that the help from the society is not the best, so sometimes they would have to adjust their work situation a lot to be able to pick up the children from school earlier, or to do separate things with these children because they can’t join their siblings on activities.
2.3.Professional and financial challenge
HCP1 (genetic counselor): There’s definitely parents who choose to take extended leaves of absence from their work to care for their children. They put their children first, if not it’s hard enough to be a working parent and having to balance your work life and your home life when you’re dealing with neurotypical children and just day to day you know demands, that’s magnified many times when you’re dealing with a child who has special needs.
HCP4 (psychologist): Some families have economic hardships so single parent families, families you know where maybe people lost their job, they’re struggling economically and it’s very difficult for them to just cope with their life let alone their child’s issues, so they’re trying to you know put a roof over their head, have money to pay the bills (…) So professional life I think people, especially in my experience, have had to maybe take leave of absence from work, they have to work part-time (…) some are actually you know quite high up in their company and they rely on a lot of paid support but in a lot of cases people can’t work full time, (…) or they might postpone applying for promotions or going after jobs that are more challenging (…)
HCP7 (nurse coordinator): I think so I mean the financials for sure, because they have to travel to centers for ongoing assessments, so that takes time away from work, it takes money to get there, you got to pay for parking, all these stuff, I mean sure there is financials and there may be a burden of care. Also just you know the toll emotionally that we just talked about, it’s so much organizing what has to be done and worrying and it’s definitely a burden of care because it’s a more complex medical diagnosis requiring ongoing assessments and changing of care.
HCP8 (genetic counselor): So obviously it’s very dependent on the medical needs of the child but I’ve had many instances where one parent ended up not going back to work after the child was born because they had so many medical needs and they just needed their mom or their dad there, and the fear of leaving your child with somebody else if they have intense medical needs (…)
HCP12 (pediatrician): I would say most of the time they continue their work but sometimes they can’t work full time because it takes time, the child needs time from the parents and I also saw that parents had problems in social psychiatric because there has been so much trouble.
HCP14 (psychologist): I think I would say financially there was definitely a few people I came across that were struggling and that did affect whether they brought them to appointments because coming several hours if they did not have a car they would have to take the train or the bus, costs a lot of money. There were couple of parents that were very proactive in seeking out any benefits that they could source and I think professionally, I mean I think in my experience, what tends to happen professionally is that one of the parents takes on a bit more of the role of caregiver, the one with a less demanding job (…)
HCP16 (speech-language therapist): Yeah I think it’s varying as well, I think some parents seem to do ok and you think waw they’re managing everything and they don’t really communicate their problems immediately. Other parents say my child is always sick, we need to be in the hospital very often, at work they are not happy with that because I always need to take time off (…) how can I combine this with my work and probably it has a financial impact as well.
HCP18 (psychologist): We see a lot of families who had to, or one of the parents, had to reduce his or her working time because it was too much otherwise to care of the child with 22q while working full time for example. So of course that has some impact financially because usually at least in (country), this is not really compensated by the pension that you get for disability pension, it doesn’t cover the time that you have. And for financial costs, in (country) we are quite fortunate that most of the medical fees are taken care by the insurance so I think that’s probably less than an issue than other things.
HCP20 (speech-language therapist): We have rather good system in (country) so you can stay home when the children are sick and get paid for it from the insurance, state insurance let’s say. But of course it still affects working life if you are away from work a lot I mean that’s a problem of course so the working place and I think it can be a lot it’s a stressful situation to have to stay home a lot for a sick kid.

#### Emotional and physical stress for the parents: when you forget to replenish your own tank

All our interviewees mentioned that children with 22q11DS’s constant need for medical attention, the numerous medical appointments, and various caregiving tasks can be physically exhausting and emotionally draining for parents and can worsen their preexisting health problems. The stress magnitude varied depending on the severity of the child’s medical situation. Moreover, many participants noted the loneliness surrounding families when caring for their children with 22q11DS due to the time and energy focus on their caretaker role and the lack of social support. According to our experts, these caregivers put themselves last on their list of care priorities.

The need for daily multitasking and the battle with time were considered by HCPs a reason why caregivers might forget to take time for self-care, particularly for the parent, generally the mother, who is often the primary caregiver. Mothers seem more affected according to many of our participants because they seem to take on more of the caregiving tasks, and to be more involved emotionally compared to dads who are more practical in their approach.

#### Unsettled family environment

The dynamics within the family unit can be deeply impacted and undergo significant changes. Parents can become very centred on the child with 22q11DS to assist them and accompany them to all their medical appointments. This results in less time for their other children. Siblings may receive less attention, leading to feelings of neglect or resentment, creating a sense of guilt for the parents. The relationships between parents may experience strain due to the overwhelming demands of caregiving and the sometimes unbalanced distribution of caregiving responsibilities, in addition to different care approaches and acceptance of the child’s condition. According to some HCPs, the impact seemed to vary depending on how strong the family bonds are and their overall well-being. One HCP reported that families are often also supportive of each other and that caring for a sibling with 22q11DS can be a life-changing experience in terms of one’s future career choice.

#### Professional and financial challenge: the burden of care

Professionally, HCPs observed that parents may face career disruptions or reduced work hours to accommodate their child’s needs, potentially affecting career progression. They recognized that many caregivers had to compromise their job aspirations, whether by giving up work, working part-time or settling for less demanding jobs, to fulfill their parenting role. One parent, the one taking up the caregiver role, seemed more affected than the other. All our participants observed that the sacrifice was not only centered on the parents’ career but also had an impact on their financial situation because they had to bring in extra help and private services to support them with their life-work balance. However, one healthcare provider noted that some families, even when in need of assistance, may hesitate to seek it due to their distrust in others caring for their child. Their perception of their child’s vulnerability might be another factor complicating their ability to manage their time effectively. Additionally, insurance and governmental coverage of absence leaves and disability pensions varied between locations.

### Areas of improvement

To support families of children with 22q11DS with their time poverty, most of our participants acknowledged that coordination of care is crucial, in addition to ensuring a good network of care that includes professional help and good communication between the different HCPs in the local and central medical facilities. They also mentioned how digitalization might play an important role (see Table 4).

**Table 4:** Solutions for time poverty

3.1.Coordination
HCP14 (psychologist): I think the most important area of improvement, is having some kind of a person that oversees everything and I don’t know what kind of person it might be, a nurse or somebody in the community or just a support worker, but for me there is something about somebody holding and tracking what’s going on with these children and this applies to a lot of kids with complex conditions and long term genetic health conditions. I sometimes feel that the parents spend a lot of time navigating their way through the system and try to work out what they need to do and in what direction they need to go (…)
HCP12 (pediatrician): I would say often we have expert teams for rare diseases and there we could have better organized coordination, to have a coordinator for the team, who’s more active, so a kind of support and also coordination to help with the many jobs of the families.
HCP2(speech-language therapist): For example, if I know that the child I’m seeing is going to see cardiology in 2 months, I put on my own schedule my speech appointment on that same day coordinated with the surgeon. If they need a hearing test and if the family’s willing to spend longer here, we do that too to decrease that burden on families. It’s a lot of navigation.
HCP13 (clinical immunologist): Two things are crucial, one is to have centers that can provide joint and coordinated care and multidisciplinary care, second education support and certainly mental health support.
HCP6 (speech-language therapist): One of the things that the 22q team does is communicate with family doctors and pediatricians in terms of what needs to be monitored in these patients, you know I wonder in places where that does not exist, who is managing or monitoring the kids from that perspective. And for families to be able to know, it’s like one stop shop for questions and concerns and being able to access what they need under the guidance of that expert team (…)
HCP8 (genetic counselor): I think I would say that the way the clinic is running, it isn’t purely multidisciplinary, it’s got a few genetic counselors and a pediatrician running the clinic. I think as long as you have the services to refer to, you don’t necessarily have to have this multidisciplinary input. You can do more like a triage or needs assessment clinic and they’re checking in once in a while (…) Of course, it’s great for the families to see experts who really know the syndrome first, but I think if you have a really good core team that can assess these kids and kind of coordinate their care, multidisciplinary care is not essential.
3.2.Digitalization
HCP7 (nurse coordinator): I think that I don’t know how much (name of doctor) and her team do virtual, at least appointments for some things now, and I would think if they’re doing some virtual, that really has you know that’s one thing out of COVID that’s made a difference that virtual sometimes is ok, you can get the blood work done at home, and sometimes that’s ok and that has opened up the doors for better access for families (…) I think more online assessments if you can do it and even preliminary online to determine if we really should see the patient, it would really support families (…)
HCP11 (pediatrician): That is one thing actually that a colleague of mine (name), a speech and language therapist who’s done a lot of early work on 22q and she’s now developing outreach by Zoom or similar to have speech therapy at home and they’re developing a web program which is actually for not specifically 22q but it’s obviously very transferable (…)
HCP8 (genetic counselor): Sometimes what happens is that (name of colleague) or I would meet with the family either by zoom like virtually or in-person if the patient is an inpatient and provide that genetic counseling and then the family has our contact and they have somebody they can talk to if they have questions as they are going along.
HCP11 (pediatrician): I think parents are always very interested in that sort of things (online services) (…) Of course in the US, there is one or two particularly educationists involved in 22q that have done a lot in helping the general community to understand the issues. If there is something like that increasingly available online that is understandable for parents as you know webinars or similar, I think that would be welcomed.
HCP2(speech-language therapist): We run a pair of programs here, it would’ve been come in person and again it’s not feasible for so many families. Now we’re running some of our parents’ programs virtually and the uptake has been much better, plus the ability to record things so that they can view things when it’s convenient for them, this has been huge (…) so the ability to do it virtually, the ability to have way more people involved because you’re not as limited in terms of physical space, the ability to record it so that they can watch it and rewatch it (…)
HCP2(speech-language therapist): We’re a downtown hospital and I would say in one respect, COVID’s slight silver lining is that we’ve actually transitioned to virtual a lot quicker than we would’ve without this pandemic happening. So for many families who would travel 2, 3, 4 h, if they don’t physically need to be seen here for whatever the reason is, then a lot of it can be done virtually. I think accessibility in that respect is improved and taken out some anxiety, both financial and time problems, because you can imagine to take time off work to travel to a hospital, to pay for parking, to have somebody take care of your other children or whatever your life back there is, that’s a huge emotional financial burden for many families.
HCP5 (ENT surgeon): When COVID was kind of in its busiest, we were doing probably 15 to 20% of our assessments were being done with Zoom. And I would say it’s really challenging specially when you’re seeing a patient for the first time, it’s ok for some postoperative things but meeting a patient and diagnosing them online and going through that discussion can be a real challenge and it’s a challenge for a number of reasons (…) The connectivity across (province) is variable, if it’s in the city it’s great but if it’s in the far north that can be sometimes a challenge. And so I would say it’s not optimal I think, my preference is to see them in person. And also looking inside their mouth through the camera, you can’t really see much (…)
3.3.Network of care
HCP3 (psychologist): So it’s good enough parenting. You don’t have to be perfect parents (…) Actually when your children are very young, they are very demanding. And when they’re older it might be easier. But in the case of 22q, it won’t get automatically. At the end it takes a village to raise a child, like the African saying. But I would say definitely in chronically ill children like 22q, you need that village but you also need a village of experts in the field of your child (…)
HCP10 (ENT surgeon): Parents can also take up some leave for instance if a child is hospitalized. There is a leave system that you can take to care for your child when they are in the hospital.
HCP18 (psychologist): We have followed families for some years and one of the sentences we hear often, caring for a child with 22q is exhausting and it can be ok for couple of months or years but years after years then it can be really exhausting. So I think there is definitely a need to develop some respite care or things that can help also the families to cope on the long run.
HCP19 (pediatrician): (…) There is also these kind of retreat things that children are being offered and the families and the siblings (…) And then if the child is diagnosed with autism, or with intellectual disability sometimes the society could offer either some extra day care or some weekend getaways or some like one week during the summer or something to relieve the family. But it’s pretty hard to get (…)
HCP1 (genetic counselor): Well for sure family and friends are important so community support right? to actual emotional support but also tangible support in terms of assisting with different tasks and different things that have to get done.
HCP4 (psychologist): In the odd case you know the child has an inherited 22q so the grandparents are helping their son or daughter and their grandchild so they can be quite involved and grandparents sometimes they help with the care, they’re the ones there when the parents are working. They’re looking after the kid or they’re taking them on the weekend or overnight. And sometimes they come to our appointments and they want to talk about what they see and they want to just hear what we have to offer.
3.4.Decentralization
HCP18 (psychologist): Definitely, there is a problem with the expertise because I don’t think every family with 22q needs to be treated by a specialist in the field of 22q because obviously it is not possible. But I think what would be the ideal solution would be to have like an expert opinion from someone who knows the syndrome and then who could be the reference for the local practitioners who are really on the daily basis with the patients and that there is really very effective communication between the two. I think that’s the most challenging part is really to also for the local practitioners to feel comfortable also seeking advice (…)
HCP6 (speech-language therapist): We also have one other option and that’s for children living in the greater area of (city) we can access (clinic) rehab and that is a rehab center where there are speech pathologists who have specific expertise working with children who have cleft related speech and velopharyngeal dysfunction (…) And we work with all of the community speech language pathologists in terms of progress, and supporting them, specially some who have not so much experience with 22q, we spend a lot of time providing guidance and support in terms of the goals.
HCP12 (pediatrician): They also have a local pediatrician where they live, and even in the town I want them to have a local pediatrician, taking care of more the acute things like infections, asthma, gastrointestinal and sleep problems, because I am more see them regularly once year or twice a year or more often earlier.
HCP11 (pediatrician): It will depend on geography, if you try to work with local services because you’re not seeing them quite frequently so you try to establish with a local community pediatrician, usually a community based pediatrician would be a good link because of their links with primary care, education and with local psychological services and they would be probably the best person in the pediatric age group to support on a much more regular basis and know who should be tapping into.

#### Coordination

The majority of experts emphasized the benefit of a coordinator to manage the care of children with 22q11DS and their families, even though this can depend on the level of proactivity of caregivers in seeking support. Our participants agreed on the role of this person in overseeing and monitoring all aspects of the comprehensive care of these children, specifically to help parents navigate the healthcare system efficiently, determining what needs to be done and in which direction to proceed. However, there were different opinions on who that person can be. Our participants agreed that their support is essential in alleviating the stress and confusion many families experience. To ensure holistic care and ongoing assessment, one HCP considered a coordination team, both for parents and HCPs, as essential to a multidisciplinary team.

#### Digitalization: the silver lining of a pandemic

Digitalization was a common topic discussed in our interviews. The COVID pandemic seemed to have played an important role in HCPs’ acceptance of and acquaintance with digital platforms. All HCPs considered digital consultations, if available, beneficial to families of children with 22q11DS and a way of minimizing the number of trips to the clinic or hospital. Virtual appointments helped families to better fit their caregiving responsibilities into their calendars, thereby reducing anxiety and stress, and enhancing the accessibility of care. The advantages of digitalization also allow parents to re-watch recorded online sessions at their discretion. Nonetheless, some healthcare providers were reluctant to fully approve of virtual meetings as there were times when physical exams and assessments may be required (i.e. first consultation) and because of the risk of a bad internet connection.

#### Network of care: it takes a village to raise a child

All of the professionals interviewed validated the importance of offering support to families who care for a child with 22q11DS. They emphasized that care should be provided formally and informally to help parents juggle daily responsibilities. Formal support means professional help and acknowledgment of the effort and time put into the caregiving process. In addition, financial coverage of leave of absence is a necessity when the parent is needed to care for the child. Respite care is also important to provide temporary relief to caregivers and support the well-being of both caregivers and care recipients (i.e. extra daycare or retreat programs for all family members). Informal support, such as respite care, can come from friends and family members, including grandparents, who, as noted by some professionals, were sometimes involved in attending medical appointments. Informal support included emotional and tangible support in terms of physical or concrete assistance as well.

#### Decentralization: a bridge of care

Some of the experts reported that it would be impossible and unnecessary for every child with 22q11DS to be treated constantly by an expert. Many of the medical issues that occur in children with 22q11DS also occur in the general pediatric population (more frequently in 22q11DS) and could be effectively managed by general HCPs. They insisted that decentralization of care ideally involves establishing a system where local practitioners receive guidance and support from experts familiar with some specific medical aspects of 22q11DS. The optimal approach would be to have experts available to consult with local healthcare providers and effective communication between them so that local practitioners feel comfortable seeking advice and can gain timely advice when faced with unfamiliar situations or treatment protocols.

## Discussion

The present study makes an important contribution to the existing literature on time management concerning care for 22q11DS by exploring the perspective of HCPs (but not directly confirmed by families’ perspective in this study). As HCPS are among the main providers of support to families of children with 22q11DS, their input is crucial in ensuring better care.

Time poverty affects not only the well-being of families but also the equity of care by challenging families’ access to services and gender inequality since primary caregivers are often mothers overwhelmed with caregiving duties. Lack of time is stressful and might cause a delay in seeking and accessing care, particularly in those situations where healthcare services are complicated to find and reach (i.e. uncoordinated care). Lack of time is an obstacle to managing caregiving responsibilities, requiring compromise from the caregivers, usually the mothers, who have to fulfill different roles (mostly unpaid work) of a parent, a wife, a household worker, and an after-school tutor and a caregiver for a child with 22q11DS.

Our study identified several reasons (e.g. disease-, family- and structural/organizational-related reasons) why families of children with 22q11DS struggle with time and different strategies to better support them. The complexity of the condition (i.e. the medical problems and their clinical unpredictability over the life course) and the family’s personal situation cannot be changed, but the coordination and centralization of care can if appropriate measures are applied.

In what follows, we discuss how coordination and centralization of care can contribute to time poverty, what are some solutions to support families with children with 22q11DS to make more efficient use of their time and the anticipated impact of these proposals.

Research shows that current practice models in RD and 22q11DS in particular, provide disjointed, fragmented care due to highly compartmentalized systems of medical management [10, 29]. The lack of coordination leads to difficulties in accessing care and additional time spent on care as parents and patients often need to coordinate their own care [10, 30–32]. Existing research shows that the integration of a coordinated care pathway for RD and 22q11DS may significantly improve the well-being of families and patients and ensure better holistic care [33, 34]. This can be done through having support from a professional to coordinate care, changing the approach of clinics and appointments (where they take place, which professionals/services are available and how they are scheduled), and improving communication through the use of technology [32]. Our participants seemed to be well aware of the fact that coordination between specialties is a very important but missing asset for better care. In a review of 11 national policies for RDs in the context of key patient needs and related to coordination, the authors concluded that Centers of Expertise (special centers joining together experts on a single or multiple RDs) or comparable programs are necessary to consolidate existing expertise and coordinate care [35]. Nonetheless, from our experts’ perspective, who are mostly working in multidisciplinary expert centers, this might not be enough and appointing a specific person or even a team to coordinate care for families with an RD is a must. In line with previous research [3], one expert, however, noted that not all HCPs are able to take on this role as they lack the necessary expertise (i.e. administration- wise etc.). According to Bambush at al., of the professionals who could manage the job best, genetic counsellors could be the best fit because they usually are in contact with most families and have extensive knowledge to guide them to the appropriate resources and support services [30].

Moreover, the tendency in RD is to bring together experts in different RDs in one specialized center in one location to treat patients in a multidisciplinary and more holistic way and to improve their quality of care. Our interviewees, however, reported that travelling to such specialized medical centers for 22q11DS was often complicated for families for different reasons. First, as most of these centers are usually located in big cities, they are distant from families living in rural areas. Second, some families may live in countries where no such centers currently exist and need to travel abroad. Third, some countries are so large that travel distances and travel time are longer.

Similarly, previous studies show that patients with 22q11DS and their families frequently voice their concerns about the burden of time and money associated with transportation for medical consultations and hospitalizations [16]. In a study with families with Huntington’s disease, distance was the first main obstacle to getting genetic support [36]. Although patients might be willing to travel longer distances to receive specialized care, it is nevertheless crucial to identify cases where centralization could jeopardize the patient’s adherence to treatment or regular appointments and thus affect equity of care [37, 38].

To avoid constant travelling for medical purposes, some of our experts discussed the importance of local HCPs skills, such as medical knowledge in speech and language therapy to ensure equity of care. The role of general practitioners and pediatricians in rural areas and small clinics in ensuring the quality of care for children and adults with RD has been emphasized by other studies as well [39]. A study conducted in Ireland, however, found that general practice largely overlooks the use of relevant RD information sources, partly due to a lack of knowledge [40]. Integrating genetics, for example, into primary care can enhance the delivery of genetic services, particularly for patients in underserved communities [41], ultimately improving equity in access to genetic diagnoses, care, and research for patients with RDs [41].

One of the emerging digital tools in the post-pandemic era are virtual case discussions, facilitated through a web-based platform called the Clinical Patient Management System. This platform enables experts from the European Reference Networks (ERNs) to provide diagnostic and therapeutic advice to treating physicians managing RDs [42]. Connecting patients with nurses, physicians, and other HCPs through tele-visits offers benefits like minimizing the need to travel to specialist clinics, lowering work absences and travel expenses, and enhancing care accessibility for vulnerable groups, including children with medical complexity (CMC) [43, 44].

Likewise, many of our experts identified digitalization as one of the main means to address both the centralization and fragmentation of care. The COVID-19 pandemic seemed to have increased our participants’ acceptance of digital platforms, with most recognizing the benefits of virtual consultations for families of children with 22q11DS, such as reducing clinic visits and easing caregiving responsibilities. However, some of our interviewees remained cautious, emphasizing the need for in-person exams and expressed concern over potential internet connection issues. In their work, A. Tozzi et al. found that 80% of families of patients with genetic diseases (including those with 22q11DS) were interested in e-health solutions for their child’s disease, 49% of them to save time [45]. Additionally, there is some evidence that for children with medical complexities (like 22q11DS), telemedicine reduces unplanned hospitalizations, healthcare service costs, and financial burdens for families while increasing caregivers’ satisfaction with care [46]. An e-resource could address other needs of carers and/or people with an RD as well: support for self-management, access to high-quality information, access to appropriate specialist services, and social support [47–50]. For example, in their study, T. Cosman et al. found that caregivers wished for a brief online intervention focused on teaching practical skills and connecting them with a peer network of support [20].

Furthermore, a network of care was mentioned by our interviewees to support patients with 22q11DS and their families. Our HCPs emphasized how stressful and energy-draining caring for a child with 22q11DS can be. This has been reported in almost all the studies with families of children with this condition [34, 51, 52]. Attributing enough time to take care of oneself and others including the child with 22q11DS overuses the caregivers’ energy and leaves them with a lot of physical and emotional stress [51]. Time poverty seems to be affecting the well-being of women more than that of men [52] because they take on most of the unpaid work (i.e. caregiving and household chores). Similar to what our HCPs mentioned, the burden of care for children with 22q11DS usually falls on the mothers [51, 53]. This raises the problem of gender equality among caregivers and necessitates appropriate support structures, for example strengthening the care infrastructure by making care services (i.e. childcare) affordable and accessible to everyone [54].

Our results suggest that 22q11DS might have an impact on the family dynamics in regards to the strain it puts on the parents as a couple and their relationship with their children. However, in a recent study in Poland with parents of children with 22q11DS, the majority of the participants surveyed denied the child’s condition affecting their relationship as a couple [16]. Additionally, research shows that 22q11DS might reinforce the relationship of parents with the affected child by spending more time with them [51]. Studies with siblings of children with 22q11DS reveal that when asked about how their life would be different if there were no 22q11DS in the family, they mentioned that they would have more attention from and time with their parents [51, 52]. Siblings have to compromise their needs for those of the family members with 22q11DS, because parents are busy taking care of their most vulnerable child [51]. Nonetheless, how the family context (i.e. parental resilience/stress) exactly affect the child with 22q11DS remains understudied [55].

From a professional and financial perspective, it was clearly visible to our HCPs that families struggled to manage their time while caring for children with 22q11DS. That meant more absences from work, difficulty maintaining a job and less money. These findings were similar to those in other studies on 22q11DS. In their recent work with Polish families with a child with 22q11DS, Walkoviak et al. reported that 50% of parents –although some were employed in the past- were unemployed due to childcare responsibilities [16]. In the literature, as caregivers of children with 22q11DS, in particular, are affected by the lack of time, particularly single parents, time flexibility (for example for working parents to attend relevant professional help) played an important role for them [56]. McMullan et al. emphasized challenges like inadequate social support and respite care, insufficient financial assistance, and a lack of awareness about available services depriving caregivers of individuals with RDs of the necessary time for self-care [57].

### Clinical recommendations

- Use specialized and multidisciplinary centers to gather top expertise on 22q11DS.
- Enhance awareness and psychoeducation of local HCPs on 22q11DS.
- Reinforce communication (i.e. with the use of digitalization) between central and local HCPs to avoid unnecessary trips and long-distance appointments for families with 22q11DS when possible. For example, having one digital medical record that can be accessed by all HCPs involved in the care of the child.
- Integrate telemedicine and digital health technologies into healthcare plans whenever useful and available.
- Assign a care coordinator or a team to coordinate appointments and follow ups and to bridge between central and local healthcare systems.
- Ensure a strong network of care is available to support families with 22q11DS, including resources to better manage their time and the impact of their time poverty (i.e. respite care, reliable information on access to care…).

#### Limitations

The majority of our participants worked at centralized expert clinics focusing on genetic diseases, including 22q11DS, where coordinated care is more common. Our recruitment was restricted to HCPs of specific regions (not including local HCPs). Two participants were involved in the broader consortium project, though not in the qualitative study itself; their connection to the project may have shaped their perspectives. We were also not able to include all medical specialties involved in the care of families of children with 22q11DS. However, given that we included the most commonly involved disciplines and those where HCPs have an overview about the types of care needed, we think that the results reflect the main aspects of care. Although we may have missed some aspects related to specific underrepresented practitioners such as some surgical specialties, there is good reasons to believe that our findings are generalizable: they depict important experts’ insights on the specificities of care for individuals with a RD and are vital for tackling the challenges faced by families of children with 22q11DS. Finally, this paper presents only health care providers’ perspectives, and families’ data were not included to confirm or contrast these views.”

## Conclusion

22q11DS is a multisystem condition requiring lifetime care for the majority of patients and the care responsibilities usually fall on parents. Time poverty for families with a child with 22q11DS result from modifiable causes such as fragmented and uncoordinated medical care, and centralized care disconnected from local care. Possible solutions include the use of digitalization in the clinical care and follow-up (i.e. increased use of telemedicine), enhancement of local professional knowledge and awareness, better communication between different HCPs, coordination and a robust support system, both formal and informal. This will enhance these families’ well-being and provide an opportunity for more equal distribution of care services among all, mitigating gender bias and addressing the needs of underserved rural populations.

## Supplementary Information

Supplementary Material 1.

## Data Availability

All data generated or analyzed during this study are included in this published article.

## Abbreviations

22q11DS: 22q11 deletion syndrome
RD: Rare disease
HCP: Healthcare professional
