# Supplementary material for: Time poverty of families of children with 22q11DS: a healthcare professional perspective
Source: BMC Pediatr. 2026 May 6;26:594. doi: 10.1186/s12887-026-06836-0 (PMC13317398; doi:10.1186/s12887-026-06836-0)
Supplement: Supplementary file 1 — Supplementary Material 1. [file 12887_2026_6836_MOESM1_ESM.pdf]

Interview Guide HCPS _ ERA net project:

<table>
<tr><td>Introduction</td><td>Explanation regarding the project and why we find it important to address this topic.</td></tr>
<tr><td>Narrative of the lived experience</td><td>I am interested in your professional experience of caring for children with 22q11 and their families.

While I have some questions to guide us along in the interview process, I am interested in hearing about anything else that you think would be useful to my understanding.

As we go through the interview, there may be questions that you would rather not answer. In this case, please feel free to "pass" on that question. Also, if any question is unclear, don't be afraid of telling me so. I will do my best to ask it more clearly.

Please be assured that your responses and all the information/data you provide are completely confidential and anonymous. Your participation in this research study is voluntary. You may withdraw at any time and for any reason without an explanation.

Then just one last reminder: there are no right or wrong answers; I am only interested in listening to what you have to say.</td></tr>
<tr><td>Interview guide:</td><td></td></tr>
<tr><td>Ice-breaker

Making space to tell their professional experience</td><td>I would like to start with a more general question regarding your professional experience. Can you briefly tell me something about your professional background?

Can you tell me a bit more about your experience of providing care for children with 22q11 and their families?
<u>Follow-up questions:</u>
(a) Can you specify your specific role in the care of children with 22q11 and their families?
(b) Are you mainly involved in the diagnosis or/and to you provide continuous/follow-up care?
(c) How much years of experience do you have & how many patients with 22q11 do you see on average each month?
(d) What do you consider the most rewarding aspects of caring for children with 22q11 and their families?
(e) What do you consider the most challenging aspects of caring for children with 22q11 and their families?

Do you face any particular difficulties when communicating the diagnosis of 22q11 to families?
<u>Follow-up questions:</u>
If so, in what sense? What do you do to address these difficulties?

Have you ever faced challenges or obstacles in optimally caring for children and their families? (multidisciplinarity)
<u>Follow-up questions:</u>
If so, when and how? What did you do?
Examples: lack of resources; parents do not adhere to treatment recommendations</td></tr>
</table>

	**Please describe a recent experience of child/family care that has made a deep impression upon you. Referring to this experience:**<br>**(a) what challenges or difficulties did you encounter?**<br>**(b) How did you handle such challenges?**<br>**(c) Please illustrate your answer with examples.**<br><br>**Let's say that you were invited to give a talk on 22q11 during a medical convention for new care providers who are interested in this pediatric field. What suggestions would you be sure to include?**
**Perceived concerns and challenges for families & patients**	**What are some of the most common reactions among parents when you inform them about their child's condition?**<br><br>**What are some of the common concerns that parents have regarding the child's present and future (when growing up) well-being? (reproductivity, future caregiver)**<br><br>**What are some of the common concerns that children and adolescents have about their present and future well-being?**<br><br>**What are according to you the most common challenges for patients and families throughout the life cycle?**<br><br>**How would you describe the impact of the child's diagnosis on families?**<br>Follow-up questions:<br>✓ Private life/ family life<br>✓ Mental health<br>✓ Physical health<br>✓ Professional life/work<br>✓ Financial situation
**Available support services**	**What types of support services exist for families raising a child with 22q11 (that you are aware of)?**<br><br>**Do you think families and children's needs for support, change while growing up?**<br>Follow-up questions:<br>If so, how do these needs change with age and what are these needs?<br><br>**How does your healthcare institution organize transition care for individuals and their families as they approach adulthood?**<br>Follow-up questions:<br>✓ Who should (ideally) be involved in the planning and coordination of this transition care<br>✓ What are some of the major challenges to this planning and coordination?<br><br>**With regard to care and support of children with 22q11, what is –according to you – the role of:**<br>✓ Parents<br>✓ Healthcare providers & multidisciplinary teams<br>✓ Teachers & schools<br>✓ Patient & family support groups

	**Do you see areas for improving the care of families and children with 22q11?**<br>Follow-up question:<br>Challenges related to multidisciplinary teams?
**Societal views**	**How do you feel the community/society at large views children with 22q11?** (e.g. ignorance, stigma, discrimination)
**Intervention tool**	We are developing an online coaching intervention, called Parents Empowering Neuro-diverse Kids Parenting Program, to improve children's wellbeing, enhance parental skills, and to reduce parental distress.<br><br>**What is your opinion regarding the availability of such a coaching program? (pro and possible cons)**<br>Follow-up questions:<br>Do you think the outbreak of COVID-19 might have changed your appraisal of such an online intervention?<br>Do you think such a program might be well-received by parents?<br>Do you think parents might prefer online or offline support interventions? Why? (role of COVID-19? Higher familiarity with online sessions?)<br>Do you think parents might prefer individual or group online sessions? Why?
**Closing questions**	**Is there anything that I have not asked that you would like to share?**
